# Comparative Properties of Two Self‐Report Measures of Avoidant/Restrictive Food Intake Disorder: Psychometric and Clinical Utility

**DOI:** 10.1002/eat.70151

**Published:** 2026-06-19

**Authors:** Rebecca Nicholls‐Clow, Arianna Prudenzi, Glenn Waller

**Affiliations:** ^1^ School of Psychology University of Sheffield Sheffield UK; ^2^ Department of Psychology University of Warwick Coventry UK

**Keywords:** Avoidant/Restrictive Food Intake Disorder (ARFID), clinical utility, eating disorders, NIAS, PARDI‐AR‐Q, psychometrics, self‐report measures

## Abstract

**Objectives:**

We compared two measures of Avoidant/Restrictive Food Intake Disorder (ARFID)—the NIAS and the PARDI‐AR‐Q to determine which demonstrates better psychometric and clinical utility.

**Methods:**

The study was pre‐registered (OSF; DOI 10.17605/OSF.IO/N8WBF). Participants (aged ≥ 18 years, BMI > 18.5) were recruited via Prolific and screened via a short written description of their fussy eating behaviors. After review, eligible participants (*N* = 156) were invited to complete a second online survey, consisting of the NIAS, PARDI‐AR‐Q, GAD‐7, PHQ‐9, CIA, and EDE‐Q7. Exploratory factor analyses were conducted separately for the NIAS and PARDI‐AR‐Q dimensions, and then on combined items. Pearson's correlations examined associations within and across both ARFID measures. Categorical agreement between measures was examined. Correlations and multiple regressions determined the extent to which ARFID subscales accounted for variance across the broader clinical outcomes.

**Results:**

All subscales within and across the NIAS and PARDI‐AR‐Q were significantly and positively correlated. The weakest association was observed between the NIAS *Picky Eating* subscale and the PARDI‐AR‐Q *Sensory Sensitivity* subscale—a pattern supported by the combined exploratory factor analysis, which yielded a four‐factor model. The NIAS and PARDI‐AR‐Q showed significant, moderate case agreement. The PARDI‐AR‐Q showed much stronger evidence of clinical validity.

**Discussion:**

Overall, the PARDI‐AR‐Q demonstrated greater clinical validity than the NIAS. The weak association between *Picky Eating* and *Sensory Sensitivity* suggests these measures capture distinct behavioral and motivational components of ARFID.

## Summary

- There has been a call for further validation of self‐report measures for ARFID in large, nonclinical samples.
- Participants completed both measures along with measures of anxiety, depression, clinical impairment, and broader restrictive eating disorder pathology.
- The PARDI‐AR‐Q is more suitable for both case identification and symptom tracking.
- Sensory‐based avoidance is operationalized differently across the NIAS (focused on behavioral manifestations) and the PARDI‐AR‐Q (focused on intrapersonal experiences).

## 1 Introduction

### 1.1 Background

Avoidant/Restrictive Food Intake Disorder (ARFID) affects 4.51% of the population (Nicholls‐Clow et al. 2024). It presents across the lifespan, although research has predominantly focused on children and adolescents (Archibald and Bryant‐Waugh 2023). ARFID symptom expressions fall into three categories: selective eating due to the sensory characteristics of food; a lack of interest in food or eating; and fear of aversive consequences (such as choking or vomiting). However, there are limited formal criteria for distinguishing these presentations. Patients can experience more than one subtype (Reilly et al. 2019; Thomas et al. 2017), and ARFID can co‐occur with symptoms of other eating disorders (Becker et al. 2020). Its impact is substantial, with severe physical and psychological symptoms, and poor health‐related quality of life (APA 2013; Hay et al. 2017).

### 1.2 Comorbidities

ARFID is associated with mental health and neurodevelopmental conditions—particularly anxiety and autistic spectrum disorders (Cooney et al. 2018; Kambanis and Thomas 2025; Nitsch et al. 2023; Richson and Zickgraf 2024). Links with low mood are less consistent and require further exploration (Aulinas et al. 2020; Richson and Zickgraf 2024). Mood and anxiety influence an individual's ability to engage in treatment and their longer‐term prognosis (Morris et al. 2025). Therefore, it is imperative to consider how these symptoms manifest alongside ARFID symptomatology.

### 1.3 Case Identification

Self‐report measures provide an efficient means of identifying ARFID. While not replacing clinical interviews, they minimize the likelihood of missed cases (Zimmerman 2024). Two frequently used self‐report measures of ARFID are the Pica, ARFID, and Rumination Disorder Interview—ARFID Questionnaire (PARDI‐AR‐Q; Bryant‐Waugh et al. 2022) and the Nine Item ARFID Screen (NIAS; Zickgraf and Ellis 2018). The PARDI‐AR‐Q is a self/parent‐report questionnaire developed from the interview form. Bryant‐Waugh et al. (2022) report good internal consistency and preliminary validity in clinical and community samples. The NIAS is a brief self‐report tool measuring the degree to which each ARFID dimension is experienced. It has good internal consistency across subscales and convergent validity with other measures of eating pathology and clinical interviews (Zickgraf and Ellis 2018).

Whilst the PARDI‐AR‐Q and the NIAS are designed to assess overlapping ARFID presentations, they differ in how these presentations are operationalized. The NIAS items primarily capture observable eating behaviors (e.g., food selectivity), whereas the PARDI‐AR‐Q includes items that assess underlying experiences associated with restrictive eating (e.g., sensory sensitivities; fear of aversive consequences). Although both measures demonstrate a three‐factor structure, these differences in item content suggest that the core domains of ARFID may be operationalized differently across the measures. Example items from both measures are provided in Supporting Information A.

### 1.4 Case Identification Challenges

Early identification of restrictive eating disorders is associated with improved prognosis and reduced risk of psychiatric and physical complications (Austin et al. 2021; Kalindjian et al. 2022). It is reasonable to expect similar patterns in ARFID, given its restrictive nature. Primary care professionals play a vital role in such early detection but often report limited training and confidence in recognizing eating disorders and in how to respond when they present (Currin et al. 2007; Hunt and Churchill 2013; Yeo and Hughes 2011).

### 1.5 Aims

It is not clear how the PARDI‐AR‐Q and NIAS perform relative to one another in non‐clinical samples, leading to a call for further evaluation (Ortiz et al. 2024). Direct comparisons are needed to clarify their psychometric properties and their clinical utility. This study addresses this gap, testing and comparing the utility of the two measures across two analytical phases. The first aims to determine and compare the psychometric properties of the two measures, and the second aims to evaluate their comparative clinical utility, to determine whether one can be advocated over the other.

### 1.6 Hypotheses

#### 1.6.1 Phase 1 (Psychometric Properties)

Hypothesis 1 *Both the NIAS and the PARDI‐AR‐Q will demonstrate acceptable internal reliability*.

Hypothesis 2 *Subscales addressing equivalent ARFID dimensions will show moderate to strong positive correlations*.

Hypothesis 3 *The NIAS and the PARDI‐AR‐Q will each yield three‐factor solutions*.

Hypothesis 4 *A combined analysis of the NIAS and the PARDI‐AR‐Q items will yield a three‐factor solution*.

#### 1.6.2 Phase 2 (Clinical Utility)

Hypothesis 5 *The NIAS will have a higher rate of positive ARFID identification*.

Hypothesis 6 *The PARDI‐AR‐Q will be more closely associated with anxiety and psychosocial impairment than the NIAS*.

## 2 Methods

### 2.1 Pre‐Registration and Ethical Clearance

The design was informed by the COnsensus based Standards for the selection of health status Measurement INstruments (COSMIN) study design checklist for patient‐reported outcome measurement instruments (Mokkink et al. 2019). The study was preregistered (Open Science Framework; 10.17605/OSF.IO/N8WBF) and received ethical clearance from The University of Sheffield.

### 2.2 Sample Size

Sample size analysis was based on the planned clinical utility analyses (Phase 2), as these involved multiple regression models and were expected to require the largest sample size. Using G*Power 3.1 (Faul et al. 2009), the multiple regression analysis required a sample of 153 to detect a medium effect size, assuming a moderate association between variables (correlation coefficient of 0.3), statistical power of 0.8, and alpha = 0.05. We aimed to recruit 300 participants for the screening survey to account for ineligible participants.

### 2.3 Participants and Recruitment

Participants were recruited via an advertisement on Prolific. Prolific is an online platform designed to connect researchers with a diverse pool of vetted participants, an increasingly used method of participant selection (Sauter et al. 2020). In previous research examining the data quality of online platforms, Prolific has produced high‐quality data—especially compared with more traditional recruitment methods such as convenience samples of students (Peer et al. 2017, 2022).

The advertisement invited individuals who self‐identified as fussy eaters to complete a short screening survey. Inclusion criteria for taking part in the screening survey were being 18+ years old, fluent in English, and currently resident in the UK. Participants were asked to take part only if they self‐identified or had been described by others as a “fussy” eater. Those who met the eligibility criteria (Supporting Information B) were invited to take part in the main body of the survey and were included in the final sample. A CONSORT diagram (Supporting Information C) details recruitment and progress.

### 2.4 Measures

Demographic and background data were collected in the screening survey (age, height [m], weight [kg], and gender). The main survey consisted of the following measures.

#### 2.4.1 PARDI‐AR‐Q (Bryant‐Waugh et al. 2022)

The PARDI‐AR‐Q is a self‐report screening tool that assesses symptoms of ARFID in line with DSM‐5 diagnostic criteria. It starts with five demographic and anthropometric questions. These are followed by 12 yes/no questions, assessing the presence of ARFID in terms of: the presence of an eating problem (2 items); low weight/faltering growth (5); nutrition deficiency (1); supplement dependence (3); and psychosocial impairment (1). The last section of the PARDI‐AR‐Q consists of 11 Likert scale items, assessing the presence and severity of each ARFID subtype (sensory‐based aversion, concern about aversive consequences, and lack of interest in eating). Identification of a possible ARFID diagnosis is based on:

- A “yes” to item 6 (“Do you think you have a problem with eating, involving avoidance or restriction of foods or your eating overall?”).
- OR a “yes” to item 7 (“Have other people [e.g., doctors, family members, significant others] said that you have a problem with eating, involving avoidance or restriction of foods or your eating overall?”).
- COMBINED with the presence of one of four other factors (significant weight loss or failure to grow, significant nutritional deficiency, dependence on enteral feeding or nutritional supplements, or psychosocial impairment).

Dimension scores (across the three ARFID profiles) and the “severity of impact” score are calculated from means of the final 11 items. High scores indicate greater severity of impact. The measure does not constitute a standalone diagnostic tool, and formal diagnosis requires structured clinical interview.

#### 2.4.2 NIAS (Zickgraf and Ellis 2018)

The NIAS is a brief self‐report questionnaire developed to screen for ARFID. Similar to the PARDI‐AR‐Q, it is not designed to diagnose ARFID, but rather to identify probable cases. The measure consists of nine items that cluster into three subscales. These assess whether a person is exhibiting restriction or avoidance relating to: *Picky Eating* (items 1–3; relating to the sensory based aversion profile of ARFID); *Appetite* (items 4–6; relating to the Lack of Interest in food or eating profile of ARFID); and/or *Fear* (e.g., choking or vomiting, items 7–9; relating to the Concern about Aversive Consequences profile of ARFID). Each item is rated on a 6‐point Likert scale from 0 (strongly disagree) to 5 (strongly agree). A higher total score (range 0–45) indicates greater avoidant/restrictive eating. Cut‐off scores (range 0–15) associated with a likely ARFID diagnosis are: picky eating ≥ 10; appetite ≥ 9; and fear ≥ 10. However, it has been suggested that this approach has a high level of incorrect case classification (Burton Murray et al. 2021). That possibility will be examined here by comparing identification rates with those of the PARDI‐AR‐Q, to develop clinical recommendations about its use.

#### 2.4.3 The Generalized Anxiety Disorder Scale (GAD‐7; Spitzer et al. 2006)

The GAD‐7 is a 7‐item self‐report measure, assessing the frequency and severity of anxiety symptoms over the past 2 weeks. Respondents rate each item on a 4‐point Likert scale (0 = not at all; 3 = nearly every day), and a total score is used.

#### 2.4.4 The Patient Health Questionnaire 9 (PHQ‐9; Kroenke et al. 2001)

The PHQ‐9 is a 9‐item self‐report measure assessing the presence and severity of depressive symptoms over the past 2 weeks (Kroenke et al. 2001; Spitzer et al. 2006). Respondents rate each item on a 4‐point Likert scale (0 = not at all; 3 = nearly every day), and a total score is used.

#### 2.4.5 Clinical Impairment Assessment Questionnaire 3.0 (CIA; Bohn et al. 2008)

The CIA is a 16‐item self‐report measure, assessing psychosocial impairment/quality of life associated with eating disorder features over the past 28 days (Bohn et al. 2008). It requires respondents to rate the extent to which their eating disorder has impacted their personal and social life and their cognitive functioning, using a 4‐point scale (0 = not at all; 3 = a lot). Higher total scores (maximum = 48) indicate greater impairment.

#### 2.4.6 Eating Disorder Examination‐Questionnaire 7‐Item Version (EDE‐Q7; Grilo et al. 2015)

The EDE‐Q7 is a brief self‐report measure of the cognitive and behavioral symptoms of eating disorders over the past 28 days. The seven items generate three subscales: dietary restraint; overvaluation of weight and shape; and body dissatisfaction (Grilo et al. 2015). Each is rated on a 0–6 scale, and higher item means indicate greater eating pathology. The EDE‐Q7 has better convergence and discrimination than the full EDE‐Q (Grilo et al. 2015; Jenkins and Davey 2020). However, it is not validated for assessing the symptoms of ARFID. In this study, the EDE‐Q7 was used to identify symptoms of other eating disorders, such as anorexia nervosa or bulimia nervosa, which would indicate incompatibility with a diagnosis of ARFID (Kambanis and Thomas 2023).

### 2.5 Procedure

The first stage of participant selection consisted of a short screening survey, hosted through Qualtrics. The study was titled “Are you a fussy eater?” “Fussy” is a term commonly used in the UK, broadly comparable to “picky eater” in the wider literature. The study description detailed that this was a preliminary survey to streamline recruitment for the main body of the research. Participants were asked: “In 150–250 words, please describe your fussy eating behaviors, including foods you avoid, why you avoid them, and how these behaviors impact your daily life and others around you.” The short essay responses were independently evaluated by two raters, using inclusion criteria (see Supporting Information B) to assess participants' engagement and comprehension, and to exclude potentially computer‐generated responses. Discrepancies were resolved through discussion, with consensus reached on final inclusion decisions. Inter‐rater reliability for the inclusion/exclusion of uncertain cases was tested using Cohen's kappa on a sample of 28 responses.

After review, those who met the inclusion criteria were sent the second survey, consisting of the PARDI‐AR‐Q and the NIAS alongside measures of broader clinical impairment (GAD‐7, PHQ‐9, CIA‐16, and EDE‐Q7). This process was conducted in two waves to ensure the necessary sample size was met. A summary of the reasons for exclusions made at each stage is provided in Supporting Information D.

Participants were compensated in line with Prolific's recommended rates of pay, receiving £1.50 for completion of the task in the screening survey, equivalent to an hourly rate of £9.00 for the estimated 10‐min duration. Participants who completed the second survey received £2.25 upon completion, equivalent to an hourly rate of £9.00 for the estimated 15‐min duration.

### 2.6 Data Analysis

Analyses were conducted using IBM SPSS Statistics (Version 29.0.2.0, IBM Corp 2022) unless stated otherwise.

#### 2.6.1 Phase 1 (Psychometric Properties)

H1. Cronbach's alpha (with an acceptable threshold of 0.70) and McDonald's omega were used to test whether the NIAS and the PARDI‐AR‐Q demonstrate acceptable internal reliability.

H2. Pearson's correlations were used to determine associations between the subscales of each measure, which were predicted to show moderate to strong positive correlations.

H3 and H4. The nine symptom items of each measure were used for factor analysis. Two separate exploratory factor analyses (EFAs) were conducted. The first identified the underlying factor structure of each measure (yielding over 10 participants per item), with the prediction that each measure would yield a three‐factor solution. The second determined the factor structure of the two measures combined (8.78 participants per item), with the prediction that a combined analysis would also yield a three‐factor solution. Factors with an eigenvalue > 1 were retained (Kaiser 1960). Scree plots were examined for each EFA, and any factors above the distinct breakpoint were retained. All three EFAs employed an Oblimin rotation (on the assumption that scales were likely to be correlated), and extracted data using Principal Axis Factoring. To further inform the factor retention for the PARDI‐AR‐Q, a parallel analysis was conducted using JASP (Version 0.96, JASP Team 2026).

#### 2.6.2 Phase 2 (Clinical Utility)

Means, ranges and reliability indices of the outcome measures were calculated for all outcome measures.

H5 predicted that the NIAS would return a higher rate of caseness than the PARDI‐AR‐Q. A chi‐squared test determined whether caseness differed between the measures. Cohen's kappa assessed the strength of agreement between the two measures.

H6 anticipated that the PARDI‐AR‐Q would be more closely associated with anxiety and psychosocial impairment than the NIAS. Pearson's correlations were conducted to evaluate the convergent validity of the ARFID scales with other clinical characteristics (anxiety, depression, eating disorder pathology, functional impairment, and BMI). Given the number of correlations, a Bonferroni correction was applied to account for multiple testing (adjusted *p* < 0.002).

Multiple regression analyses were conducted to determine the extent to which the six ARFID dimension subscales across the two measures accounted for variance in anxiety, depression, functional impairment scores, BMI, and broader disordered eating pathology. Multicollinearity was assessed using variance inflation factors.

## 3 Results

Agreement on whether to include borderline participants based on their essay responses was moderate (*κ* = 0.47, SE = 0.13, *p* = 0.003; observed percentage agreement = 71.43%). The final sample after the screening survey (*N* = 156) consisted of 87 (55.8%) females and 69 (44.2%) males. Mean age was 39.77 years and mean BMI was 25.74 (Table 1). Descriptive characteristics of the participants who completed the screening survey can be found in Supporting Information E.

**TABLE 1:** Sample demographics.

Characteristic	Minimum	Maximum	Mean	SD
Age	18	75	39.77	12.47
Height (m)	1.56	2.00	1.71	0.107
Weight (kg)	44.50	183.0	75.47	18.18
BMI	18.73	54.64	25.74	5.38

	Male *n* (%)	Female *n* (%)
Gender	69 (44.2%)	87 (55.8%)

### 3.1 Phase 1 (Psychometric Properties)

H1, which predicted that both the NIAS and the PARDI‐AR‐Q would demonstrate acceptable internal reliability, was supported. Good to excellent internal consistency was demonstrated across the NIAS scales—*Picky Eating* (*α* = 0.86, *ω* = 0.86), *Fear* (*α* = 0.89, *ω* = 0.90), and *Appetite* (*α* = 0.91, *ω* = 0.91). The PARDI‐AR‐Q scales similarly demonstrated good to excellent consistency—*Sensory‐based Avoidance* (*α* = 0.83, *ω* = 0.84), *Concern about Aversive Consequences* (*α* = 0.87, *ω* = 0.87), and *Lack of Interest* (*α* = 0.80, *ω* = 0.80).

In line with H2, all subscales within the two measures were significantly and positively correlated (*p* < 0.001). The level of association was *r* = 0.47–0.60, indicating a quarter to a third of common variance.

H3 predicted that the NIAS and the PARDI‐AR‐Q would each yield a three‐factor solution. *NIAS*. The assumptions of the EFA for the NIAS were checked to ensure the correlation matrix was factorable. Bartlett's test of sphericity indicated that correlations between items were sufficiently large for factor analysis, *χ* ^2^ (36) = 967.075, *p* < 0.001. The Kaiser–Meyer–Olkin (KMO) measure of sampling adequacy was acceptable at 0.840 (Kaiser 1974). Eigenvalues and scree plot indicated that three factors should be retained. Each of those factors had three items, where the loading of each item was greater than 0.6 and none of the items had a significant cross‐loading with other factors. The three factors aligned exactly with the existing NIAS scales and were labeled accordingly as *Picky Eating* (items 1, 2, 3), *Fear* (items 7, 8, 9), and *Appetite* (items 4, 5, 6).

*PARDI‐AR‐Q*. Bartlett's test of sphericity confirmed that correlations between items were sufficiently large for factor analysis, *χ* ^2^ (36) = 732.84, *p* < 0.001. The KMO value (0.856) confirmed suitability for factor analysis. Using eigenvalue > 1 indicated that two factors should be retained. However, a visual inspection of the scree plot and the results from the parallel analysis supported a three‐factor model. We proceeded with that three‐factor solution, as it best aligned with the established structure of the PARDI‐AR‐Q (Bryant‐Waugh et al. 2022). The two factors informed by the Kaiser criterion demonstrated salient loading of 0.50 or higher for all items, with the third factor showing similar loadings apart from one loading of 0.49. No items showed problematic cross‐loadings. The three factors matched existing PARDI‐AR‐Q scales and were labeled accordingly as *Sensory‐based Avoidance* (items 24, 25, 26), *Concern about Aversive Consequences* (items 30, 31, 32), and *Lack of Interest* (items 27, 28, 29).

H4 predicted that a combined analysis of the NIAS and the PARDI‐AR‐Q would also yield a three‐factor solution. Bartlett's test of sphericity, *χ* ^2^ (153) = 1968.09, *p* < 0.001 demonstrated that item correlations were sufficient for factor analysis, supported by the KMO (0.906). For the combined analysis, no restrictions were imposed, and factor extraction was guided by the Kaiser criterion. Based on eigenvalues > 1 and visual inspection of the scree plot, four factors were retained. Across those four factors, two had three items with salient loadings, and two had six such items each (all > 0.48), as summarized in Table 2. Factor 1 was labeled as *Sensory‐based Avoidance* (three items), Factor 2 as *Concern about Aversive Consequences* (six items), Factor 3 as *Lack of Interest* (six items), and Factor 4 as *Picky Eating* (three items).

**TABLE 2:** EFA results for the combined analysis of the NIAS and the PARDI‐AR‐Q.

	Factor 1: Sensory‐based Avoidance	Factor 2: Concern about Aversive Consequences	Factor 3: Lack of Interest	Factor 4: Picky Eating
% Variance explained	7.40	9.99	47.14	9.61
Eigenvalue	1.33	1.80	8.49	1.73
Cronbach's *α*	0.86	0.91	0.90	0.86
McDonald's *ω*	0.85	0.91	0.90	0.85
Measures/items				
NIAS				
Q1	0.08	0.04	0.01	0.81
Q2	0.12	0.01	0.04	0.83
Q3	−0.06	−0.08	0.02	0.74
Q4	−0.17	−0.02	0.79	0.20
Q5	−0.15	−0.06	0.81	0.09
Q6	−0.13	−0.20	0.82	−0.01
Q7	−0.13	−0.79	0.10	0.09
Q8	−0.16	−0.83	−0.10	0.25
Q9	−0.16	−0.63	0.27	0.07
PARDI‐AR‐Q				
Q24	0.51	−0.11	0.14	0.29
Q25	0.59	−0.16	0.05	0.29
Q26	0.48	−0.25	0.12	0.21
Q27	0.25	0.12	0.78	−0.04
Q28	0.38	−0.00	0.52	0.11
Q29	0.13	−0.10	0.63	−0.07
Q30	0.25	−0.75	0.05	−0.07
Q31	0.06	−0.81	0.07	−0.06
Q32	0.29	−0.67	−0.02	−0.07

### 3.2 Phase 2 (Clinical Utility)

H5. Table 3 shows the number of participants classified as meeting the threshold for a possible ARFID diagnosis across both the PARDI‐AR‐Q and the NIAS. The measures demonstrated moderate agreement in ARFID case classification. A Chi‐square test confirmed a significant association between the two measures, *χ* ^2^ (1, *N* = 156) = 34.61, *p* < 0.001, with a moderate to large effect size (*φ* = 0.47), exceeding the conventional threshold for a moderate effect (*φ* = 0.30; Cohen 1988). Cohen's *κ* showed a moderate level of agreement beyond chance, *κ* = 0.47, *p* < 0.001, consistent with conventional benchmarks (Landis and Koch 1977). All three presentations of ARFID were evident across the sample.

**TABLE 3:** Agreement between the PARDI‐AR‐Q and the NIAS case classifications.

		NIAS		Total
		Negative	Positive	
PARDI‐AR‐Q	Negative	50	18	68
	Positive	23	65	88
Total		73	83	156

H6. The mean scores on all clinical measures (Table 4) were below the established cut‐offs for moderate to severe symptomatology, as would be expected in a non‐clinical sample. All measures demonstrated excellent internal consistency, supporting suitability for use in the present analyses.

**TABLE 4:** Means, ranges, and reliability indices of the GAD‐7, PHQ‐9, EDE‐Q7, and CIA (outcome measures) and Pearson's correlations between ARFID subscale scores with the outcome measures.

	Mean score (SD)	Range	Clinical cutoff	Cronbach's alpha	McDonald's omega
GAD‐7	7.39 (5.80)	0–20	≥ 10	0.929	0.930
Minimal anxiety			0–4		
Mild anxiety			5–9		
Moderate anxiety			10–14		
Severe anxiety			15+		
PHQ‐9	8.45 (6.80)	0–27	≥ 10	0.925	0.927
Mild depression			5		
Moderate depression			10		
Moderately severe depression			15		
Severe depression			≥ 20		
EDE‐Q7 (total)	2.68 (1.73)	0–6	≥ 3.64^a^		
EDE‐Q7 dietary restraint				0.947	
EDE‐Q7 overvaluation of shape and weight				0.727	
EDE‐Q7 body dissatisfaction				0.941	0.941

	Correlations			
	GAD‐7	PHQ‐9	EDE‐Q7	CIA
NIAS				
Picky Eating	**0.366** **	**0.298** **	0.139	**0.379** **
Appetite	**0.523** **	**0.480** **	0.151	**0.498** **
Fear	**0.409** **	**0.366** **	0.159*	**0.481** **
PARDI‐AR‐Q				
Sensory	**0.540** **	**0.516** **	0.**328** **	**0.645** **
Lack of Interest	**0.594** **	**0.575** **	**0.347** **	**0.503** **
Concern	**0.427** **	**0.444** **	0.212**	**0.588** **

*Note:* Bold values indicate results that remained statistically significant following adjustment for multiple comparisons.

Abbreviations: CIA, Clinical Impairment Assessment; EDE‐Q7, Eating Disorder Examinations Questionnaire 7 item version; GAD‐7, Generalized Anxiety Disorder questionnaire 7 item version; PHQ‐9, Patient Health Questionnaire 9 item version; SD, standard deviations.

^a^ Clinical cut off based on Bang et al. (2023).

* *p* < 0.05.

** *p* < 0.001.

Table 4 (correlations) shows that all three ARFID subscales from the PARDI‐AR‐Q were at least moderately associated with anxiety, depression and clinical impairment (all *r* ≥ 0.3), with several large associations (*r* ≥ 0.5). These associations were generally weaker for the NIAS, especially in relation to the PHQ‐9. In contrast, correlations with the EDE‐Q7 were comparatively small, consistent with ARFID being distinct from other eating disorders (e.g., anorexia nervosa or bulimia nervosa).

Table 5 (multiple regressions) shows that NIAS subscales were not significantly associated with anxiety, depression, functional impairment or broader eating disorder pathology. In contrast, the PARDI‐AR‐Q dimensions of *Sensory‐based Avoidance* and *Lack of Interest* were consistently associated with more of the clinical measures (variance accounted for: anxiety = 40%; depression = 37%), and all three PARDI‐AR‐Q scales were associated with clinical impairment (50% of variance). However, only PARDI‐AR‐Q *Lack of Interest* was associated with the EDE‐Q7 score (13% of variance). The NIAS *Appetite* subscale showed the only association with BMI across both measures, explaining 9% of variance. Multicollinearity diagnostics indicated acceptable levels, with all variance inflation factors below 3, well under commonly cited thresholds for concern (Kim 2019).

**TABLE 5:** Multiple regression analyses predicting clinical outcomes from subscales of the PARDI‐AR‐Q and the NIAS.

	GAD‐7	PHQ‐9	CIA	BMI	EDE‐Q7
Model statistics					
Adjusted *R* ^2^	0.404	0.373	0.516	0.093	0.133
*F* (df = 6149)	18.52	16.36	28.91	3.64	4.97
*p*	< 0.001	< 0.001	< 0.001	< 0.002	< 0.001

	*β*	*t*	*p*	*β*	*t*	*p*	*β*	*t*	*p*	*β*	*t*	*p*	*β*	*t*	*p*
NIAS															
Picky Eating	−0.09	−0.11	0.911	−0.07	−0.83	0.410	−0.05	−0.68	0.498	0.121	1.22	0.226	−0.05	−0.53	0.598
Fear	0.06	0.63	0.531	−0.01	−0.13	0.893	0.03	0.35	0.730	0.056	0.46	0.648	0.06	0.50	0.615
Appetite	0.16	1.50	0.135	0.13	1.25	0.231	0.07	0.71	0.477	−0.465	−3.65	< 0.001**	−0.22	1.80	0.073
PARDI‐AR‐Q															
Sensory	0.26	2.78	0.006**	0.25	2.61	0.01**	0.351	4.20	< 0.001**	0.163	1.42	0.159	0.19	1.73	0.086
Concern	0.02	0.22	0.827	0.13	1.28	0.201	0.238	2.73	0.007**	−0.043	−0.36	0.719	−0.01	−0.05	0.962
Lack of Interest	0.29	2.84	0.005**	0.30	2.84	0.005**	0.236	2.54	0.12	0.10	0.75	0.458	0.39	3.13	0.002**

** *p* < 0.001.

## 4 General Discussion

This is the first study to compare the psychometric properties and clinical validity of the NIAS and PARDI‐AR‐Q in non‐clinical adults, in order to provide guidance regarding the optimum measure to identify and monitor cases of ARFID.

### 4.1 Relationship of Findings to the Wider Literature

#### 4.1.1 Psychometric Properties of the Measures

The first phase indicated acceptable reliability and confirmed both measures' construct validities, supporting Hypotheses 1 and 2. Hypothesis 3 was also supported, as each measure yielded a three‐factor structure. However, contrary to Hypothesis 4, a combined EFA returned a four‐factor solution, with distinct *Picky Eating* (NIAS) and *Sensory‐Based Avoidance* (PARDI‐AR‐Q) subscales.

The combined analysis shows that these measures assess some similar ARFID features (loss of interest in food; fear of negative consequences), and that all the scales are independently cohesive, consistent with Hypothesis 1 (Bryant‐Waugh et al. 2022; Melisse et al. 2025; Zickgraf and Ellis 2018). However, the distinction between the *Picky Eating* and *Sensory‐based Avoidance* scales reflects the different conceptualizations of the two measures. The NIAS *Picky Eating* items measure observable behaviors, while the PARDI‐AR‐Q *Sensory‐Based Avoidance* items address internal experience. While Zickgraf et al. (2025) suggest reformulating ARFID to include both the *motivation* and the *behavioral* manifestations of picky eating into a single “selective eating” construct, the failure of the *Picky Eating* and *Sensory‐Based Avoidance* to coalesce into a single factor suggests that this proposal might be unsuccessful.

#### 4.1.2 Clinical Validity of the Two Measures

In the second phase, Hypothesis 5 was not supported, as the PARDI‐AR‐Q identified more cases than the NIAS. The PARDI‐AR‐Q has been suggested to more accurately identify adults with ARFID in community samples (Ortiz et al. 2024), indicating that it is more sensitive to the clinical features of ARFID in non‐treatment‐seeking populations, possibly because the PARDI‐AR‐Q and EDE‐Q7's correlation reflects overlap at the level of broader disordered eating. Despite DSM‐5 stating that ARFID cannot co‐occur alongside other restrictive eating disorders, recent evidence suggests that this is incorrect (Zickgraf et al. 2025).

Multiple regressions supported Hypothesis 6, with the PARDI‐AR‐Q more closely associated with anxiety, depression and broader clinical impairment. *Lack of Interest/Appetite* across both measures showed the strongest associations with anxiety, despite previous suggestions that this would be the case for *Concern About Aversive Consequences* (Menzel and Perry 2024; Norris et al. 2018). The NIAS showed more limited clinical validity. The NIAS *Appetite* subscale was the only one that was associated with BMI, in keeping with research linking the *Lack of Interest* presentation to low weight status (Norris et al. 2018; Thomas et al. 2017). Other NIAS scales did not contribute independent variance to clinical measures, over and above the PARDI‐AR‐Q.

### 4.2 Research Strengths and Limitations

A strength of this study is that it evaluated the PARDI‐AR‐Q and NIAS with a sufficient sample size to reach robust conclusions in most analyses. As suggested by Ortiz et al. (2024), it provides a direct comparison of the properties of the two measures in a large, adult, nonclinical sample. It also stresses the importance of subscale‐level analysis of the measures.

The study also has limitations. First, the non‐clinical adult sample means that the findings cannot be assumed to generalize to younger, clinical samples. Further research into these populations is needed. The potential impact on generalizability of the use of the online recruitment platform, Prolific, must be considered. Whilst Prolific has been shown to provide relatively diverse and high‐quality samples (Peer et al. 2022), online samples might differ from treatment‐seeking participants, and participation is restricted to individuals with internet access. In addition, ethnicity data were not collected, limiting the extent to which the diversity of the sample can be characterized and restricting the conclusions that can be drawn. This limitation is further compounded by the sample being recruited solely from the UK, as the findings might not generalize to other countries and cultural presentations of ARFID. For example, it is acknowledged that the use of the term “fussy eating” might be a limitation of the research. Used in the screening process, “fussy eating” is a colloquial term commonly used in the UK. Although conceptually similar to “picky eating,” differences in interpretation may have influenced participant responses and potentially shaped the composition of the sample.

It is also noted that self‐report measures alone cannot confirm a diagnosis of ARFID. The next stage in clinical validation needs to be establishing associations with diagnostic interviews, along with determining which measures reflect clinical recovery across therapies for ARFID.

Finally, the combined exploratory factor analysis was conducted with 18 items and a sample of 156 participants (participant to item ratio 8.78:1) which falls slightly below the recommendation of 10:1 (Everitt 1975). While such guidelines are not absolute, the sample size might limit the stability and replicability of the combined factor analysis. Replication in larger samples is warranted.

### 4.3 Clinical Implications

This study offers guidance for clinicians and researchers who need practical tools to identify potential ARFID cases and to measure progress and outcomes. The PARDI‐AR‐Q had greater clinical sensitivity than the NIAS. Therefore, we concur with Ortiz et al. (2024) when a single measure is required for case identification, the PARDI‐AR‐Q is preferable. Its wider clinical validity relative to comorbid conditions supports its use as a measure of change throughout treatment. Progress on these scales should be considered particularly early in therapy, given that early change in eating disorders (and other disorders) is a strong predictor of positive outcomes (Chang et al. 2021). Specifically, PARDI‐AR‐Q subscale items (items 24–32) could be used to monitor progress, while the full scale could be used to measure outcomes from the beginning to the end of therapy.

Clinicians should not assume that the NIAS and PARDI‐AR‐Q measure the same constructs fully. If the behavioral element of *Picky Eating* (NIAS) is clinically important in the individual case (e.g., very underweight), then the NIAS is worthy of consideration. However, if the intrapersonal element of *Sensory‐Based Avoidance* is key, then the PARDI‐AR‐Q is a more meaningful measure. If both are relevant, then the *Picky Eating* subscale could be used alongside the PARDI‐AR‐Q.

### 4.4 Conclusions

While the NIAS might reflect restrictive eating, the PARDI‐AR‐Q provides the most clinically relevant measure of both caseness and symptom tracking. Future research is required to support these findings with clinical groups.

## Author Contributions

**Rebecca Nicholls‐Clow:** conceptualization, writing – original draft, data curation, formal analysis, methodology, project administration, writing – review and editing. **Arianna Prudenzi:** supervision, writing – review and editing. **Glenn Waller:** conceptualization, formal analysis, methodology, supervision, writing – review and editing.

## Lived Experience Involvement Statement

No specific efforts were undertaken to involve persons with lived experience in the study design or execution, or in the preparation of this manuscript.

## Funding

The authors have nothing to report.

## Conflicts of Interest

The authors declare no conflicts of interest.

## Supporting information


**Supporting Information A:** Example items and subscale mapping for the PARDI‐AR‐Q and the NIAS.
**Supporting Information B:** Inclusion criteria used to assess mini essay responses.
**Supporting Information C:** CONSORT flowchart of participants.
**Supporting Information D:** Reasons for participant exclusion.
**Supporting Information E:** Sample demographics from the pre‐screening sample.

## Data Availability

The data that support the findings of this study are available from the corresponding author upon reasonable request.
