# Supplementary material for: Comparative Properties of Two Self‐Report Measures of Avoidant/Restrictive Food Intake Disorder: Psychometric and Clinical Utility
Source: Int J Eat Disord. 2026 Jun 19;59(10):2226–36. doi: 10.1002/eat.70151 (PMC13655934; doi:10.1002/eat.70151)
Supplement: Supplementary file 1 [file EAT-59-2226-s001.docx]

**Supplementary Material A**

Example Items and Subscale Mapping for the PARDI-AR-Q and the NIAS

| Measure | Subscale | ARFID Profile | Example Item |
| --- | --- | --- | --- |
| PARDI-AR-Q | Sensory Based Aversion | Sensory Based Aversion | Over the past month, have you been particularly sensitive to the appearance of food, which has put you off eating any food or trying any new food (for example, if a food does not look ‘right’, such as burnt end of chips/friends, broken biscuits/cookies, or being the ‘wrong’ colour) |
| PARDI-AR-Q | Concern About Aversive Consequences | Concern About Aversive Consequences | Over the past month have you been avoiding or restricting the amount or type of food you eat because you were afraid that something bad might happen, like being sick, choking, having an allergic reaction, or being in pain? |
| NIAS | Picky Eating | Sensory Based Aversion | (To what extent do you agree with the following statements…) I am a picky eater. |
| NIAS | Fear | Concern About Aversive Consequences | (To what extent do you agree with the following statements…) I eat small portions because I am afraid of GI discomfort, choking, or vomiting. |

**Supplementary Material B**

*Inclusion Criteria Used to Assess Mini Essay Responses*

| Criteria Type | Eligible | Ineligible |
| --- | --- | --- |
| BMI | Over 18.5 | Under 18.5 |
| Content *(If criteria 1 is met)* | Includes ONE or more of the following:  E1. Mentions fussy eating directly.  E2. Mentions sensory based avoidance of any food.  E3. Mentions a lack of interest in food or eating.  E4. Mentions a fear of a food/eating/a consequence of eating  E5. Mentions restriction with no reason (i.e. no weight loss goals)  E6. Mentions weight loss or growth issue due to food avoidance  E7. Mentions a negative impact on their social life or mental health because of food. |  |
|  |  |  |

**Supplementary Material C**

*CONSORT Flowchart of Participants*

**Supplementary Material D**

*Reasons for Participant Exclusion*

| Reason for exclusion | Number of participants |
| --- | --- |
| Suspected AI | 23 |
| Weight loss/health-related motivation for food avoidance | 55 |
| No fussy eating described | 49 |
| Data error | 25 |
| Moral motivation for food avoidance | 9 |
| BMI too low | 6 |
| Preferences described as opposed to fussy eating behaviour | 6 |
| Budgeting concerns over food | 2 |
| Cleanliness concerns over food | 1 |
| General anxiety (e.g. eating in public) | 1 |
| Total | 177 |

**Supplementary Material E**

Sample Demographics from the Pre-Screening Sample

| Characteristic | Minimum | Maximum | Mean | SD |
| --- | --- | --- | --- | --- |
| Age | 18 | 76 | 40.11 | 12.65 |
| Height (m) | 1.3 | 2.2 | 1.7 | .11646 |
| Weight (kg) | 44 | 182 | 76.87 | 18.56 |
| BMI | 17.19 | 54.35 | 26.53 | 5.9 |
|  | Male *n* (%) | Female *n* (%) |  |  |
| Gender | 191 (51.6 %) | 179 (48.4%) |  |  |
